# Effects of thyroxin and donepezil on hippocampal acetylcholine content and syntaxin-1 and munc-18 expression in adult rats with hypothyroidism

**DOI:** 10.3892/etm.2014.1487

**Published:** 2014-01-16

**Authors:** NAN WANG, YAOJUN CAI, FEN WANG, XIANZHONG ZENG, XUEMEI JIA, FANGBIAO TAO, DEFA ZHU

**Affiliations:** 1 Department of Endocrinology, Anhui Geriatric Institute, The First Affiliated Hospital, Anhui Medical University, Hefei, Anhui 230022, P.R. China; 2 Comprehensive Laboratory, College of Basic Medicine, Hefei, Anhui 230032, P.R. China; 3 College of Public Hygiene, Anhui Medical University, Hefei, Anhui 230032, P.R. China

**Keywords:** hypothyroidism, hippocampus, thyroxin, donepezil, acetylcholine, syntaxin-1, munc-18

## Abstract

Adult-onset hypothyroidism induces various impairments in hippocampus-dependent cognitive function, in which numerous synaptic proteins and neurotransmitters are involved. Donepezil (DON), an acetylcholinesterase inhibitor, has been shown to be efficient in improving cognitive function. The aim of the present study was to investigate the effects of adult-onset hypothyroidism on the expression levels of the synaptic proteins syntaxin-1 and munc-18, as well as the content of the neurotransmitter acetylcholine (ACh) in the hippocampus. In addition, the study explored the effects of thyroxin (T4) and DON treatment on the altered parameters. The study involved 55 Sprague-Dawley rats that were randomly divided into five groups: Control, hypothyroid (0.05% 6-n-propyl-2-thiouracil; added to the drinking water), hypothyroid treated with T4 (6 μg/100 g body weight once daily; intraperitoneal injection), hypothyroid treated with DON (0.005%; added to the drinking water) and hypothyroid treated with a combination of the two drugs (6 μg/100 g T4 and 0.005% DON). The concentration of ACh was determined in the homogenized hippocampus of each animal by alkaline hydroxylamine colorimetry. The protein levels of syntaxin-1 and munc-18 were determined by immunohistochemistry. The results showed that the content of ACh in the hippocampi of the hypothyroid rats was significantly decreased compared with that in the controls and that T4 monotherapy and DON administration restored the ACh content to normal values. In the hippocampi of the hypothyroid group, munc-18 was expressed at significantly lower levels, while the expression levels of syntaxin-1 were increased compared with the levels in the control group. Treatment with T4 alone restored the expression of syntaxin-1 but failed to normalize munc-18 expression levels. The co-administration of T4 and DON returned the munc-18 levels to normal values. These observations indicate that adult-onset hypothyroidism induces alterations in the levels of munc-18, syntaxin-1 and ACh in the hippocampus. Syntaxin-1 and ACh levels were restored by T4 monotherapy while munc-18 levels were not. In addition, the co-administration of T4 and DON resulted in more effective restoration than either alone. The thyroid hormone has a direct effect on metabolism of hippocampal ACh in adult rats and DON is helpful for treatment of synaptic protein impairment induced by hypothyroidism.

## Introduction

Adult-onset hypothyroidism leads to hippocampus-dependent cognitive dysfunction, in which several neurotransmitter systems and synaptic proteins are involved (1–4). Neurotransmitters, which are stored in synaptic vesicles in presynaptic neurons, are the material foundation of synaptic transmission. The release of a neurotransmitter requires the assistance of a variety of synaptic proteins (5,6). Acetylcholine (ACh), which is involved in learning and memory, is a significant neurotransmitter in the brain and has a close relationship with thyroid hormones (THs) (2). Studies using gene recombination technology have revealed that the synaptic proteins syntaxin-1 and munc-18 are involved in the release of ACh in mouse brains (7,8). Syntaxin-1, abundantly expressed in the presynaptic membrane, has been implicated in synaptic vesicle docking, which is the initial association of synaptic vesicles with the plasma membrane (9). Munc-18 is a neuronal protein that binds tightly to an N-terminal peptide sequence in syntaxin-1 and accelerates the fusion of neurotransmitter-containing synaptic vesicles and the plasma membrane (10).

Thyroxin (T4) replacement therapy is a validated treatment for hypothyroidism. However, for patients with cognitive dysfunction, the data regarding treatment with T4 are ambiguous. In certain cases, T4 replacement therapy has been found to restore the levels of triiodothyronine (T3), T4 and thyroid-stimulating hormone (TSH) and fully remedy molecular impairments exhibited in the hypothyroid brain. However, in other patients, these effects were not observed (11,12). In addition, the concentrations of Ca^2+^/calmodulin-independent protein kinase (CaMKII), neurogranin, SNAP-25 and calmodulin, in which changes were induced by hypothyroidism, have been found to return to basal levels following T4 replacement therapy. However, the levels of protein kinase C-γ and synaptotagmin-1 in the hippocampus were not restored in adult hypothyroid rats receiving T4 replacement therapy (11,13,14). These observations indicate that it is necessary to identify new alternative therapeutic methods for treating hypothyroidism.

Donepezil (DON), a potent acetylcholinesterase (AChE) inhibitor, has demonstrated clinical efficacy, increasing the levels of ACh at synapses and thereby ameliorating memory and cognition impairments (15). At present, DON is widely administered for the treatment of mild cognitive impairment (16,17). In the present study, the ability of DON to treat the neurocognitive parameter impairments in hypothyroidism was investigated. Therefore, the expression levels of munc-18 and syntaxin-1, as well as the ACh content, were observed in the dorsal hippocampi of rats with adult-onset hypothyroidism. In addition, the efficacies of T4 and DON in the treatment of the altered parameters were investigated.

## Materials and methods

### Animals

Three-month-old adult male Sprague-Dawley rats (n=55) were obtained from the Nanjing Experimental Animal Center (Nanjing, China). The animals were maintained at room temperature under natural light-dark cycle conditions and received a standard rodent diet and water *ad libitum*. The body weight (BW) of the rats was recorded weekly to monitor growth inhibition, which is a marker of hypothyroidism. Procedures involving animals and their care were performed in accordance with the Animal Care and Use Committee of Anhui Medical University (Hefei, China).

The rats were randomly classified into five groups: Control, hypothyroid, hypothyroid receiving T4 replacement therapy, hypothyroid receiving DON therapy and hypothyroid receiving T4 plus DON therapy. Hypothyroidism was induced in the hypothyroid group (Hypo group) by adding 6-n-propyl-2-thiouracil (PTU; Sigma-Aldrich, St. Louis, MO, USA) to the drinking water at a concentration of 0.05% (w/v) for six weeks (n=11). The DON group was treated with PTU for six weeks, as described for the Hypo group and from the fifth week, 0.005% (w/v) DON (Sigma-Aldrich) was added to the drinking water every day for two weeks (n=11). The T4 group was treated with PTU for six weeks, as described for the Hypo group and from the fifth week, T4 (dissolved in saline solution, 6 μg/100 g BW) was injected intraperitoneally for two weeks to restore the hypothyroid animals to euthyroid status (n=10). The T4 plus DON group (T4 + DON group) was treated according to the same protocol as the T4 group for six weeks with the modification that 0.005% (w/v) DON was added to the drinking water from the fifth week (n=11). The control group (C group) was administered the same volume of saline solution for six weeks (n=12).

### Thyroid hormones

Rats were anesthetized using chloral hydrate (350 mg/kg BW), following the delivery of the final dose. Next, blood collected from the abdominal aorta (1.5 ml), underwent centrifugation at 14,000 × g for 15 min. Prior to subsequent analysis, the serum was rapidly frozen at −20ºC. T3 and T4 serum concentrations were obtained using a radioimmunoassay kit (North Institute of Biological Technology, Beijing, China). The detection ranges of the assay were: T3, 0.92–2.78 nmol/l; T4, 58–140 nmol/l; and TSH, 0.5–4.7 μIU/ml

### Tissue preparation

Rats were sacrificed and the brains were dissected on ice following blood collection. For immunohistochemistry, the right brains were isolated and then fixed in 4% paraformaldehyde for 7 days at 4ºC. The hippocampus from the left brain was stored at −80ºC prior to determining the content of ACh.

### Determination of ACh content

Rats were sacrificed, and the hippocampus was separated. After weighting, 9 ml of normal saline was added to 1 g of hippocampus, followed by homogenation in a glass homogenizer. ACh content in hippocampus homogenates was measured by the modified method of Hestrin (18) to compare the amounts of this key neurotransmitter in the hippocampus among the groups, as previously described. Briefly, 0.2 ml supernatant was mixed with 0.35 ml distilled water followed by addition of 0.05 ml calabarine sulfate (1.5 mmol/l) and 0.2 ml trichloroacetic acid (1.84 mol/l). The mixture was centrifuged at 5,000 × g for 5 min. Next, 0.1 ml ultimate supernatant was added to 0.1 ml alkaline hydroxylamine hydrochloride (equal volumes of 2.0 mol/l hydroxylamine hydrochloride and 3.5 mol/l sodium hydroxide), incubated at room temperature for 15 min and reacted with 0.05 ml HCl (4.0 mol/l) and 0.05 ml ferric chloride (0.37 mol/l, containing 0.1 mol/l HCl). Next, 0.2 ml medium and tissue homogenates were spotted in duplicate onto 96-well microplates. Physostigmine (1.5 mmol/l) was added to the reaction mixture to inhibit the activity of AChE. Following an additional 2 min incubation, the intensity of the brown ferric complex was read at 540 nm on a Take3™ plate reader (BioTek Instruments Inc., Winooski, VT, USA). ACh levels were expressed in micrograms per milligram of hippocampal protein (μg/mg prot).

### Protein assay

Protein in the hippocampal homogenates was detected using a BCA Protein Assay kit (Thermo Fisher Scientific, Waltham, MA, USA) according to the manufacturer’s instructions.

### Immunohistochemistry

The fixed right hemispheres were embedded in paraffin and sectioned coronally using a microtome to produce 6-μm-thick sections. Five sections (1/20 serial sections) of the dorsal hippocampus were selected from each rat and mounted on polylysine-coated slides. Following deparaffinization, each section underwent antigen retrieval, by heating in 10 mM citrate buffer (pH 6.0) at 100ºC for 10 min. Non-specific binding was blocked using 5% normal goat serum in PBS. The sections were then incubated with mouse anti-munc-18 (1:200; BD Biosciences, Franklin Lakes, NJ, USA) or rabbit anti-syntaxin-1 (1:400; Millipore, Temecula, CA, USA) primary polyclonal antibodies at 37ºC for 1 h and overnight at 4ºC. Next, sections were washed in PBS, incubated with biotinylated secondary antibody [rabbit anti-mouse or goat anti-rabbit IgG (Bioss-Bio Ltd., Beijing, China)] for 15 min at 37ºC and washed again in PBS. Sections were incubated further with HRP for 10 min at 37ºC, washed in PBS and colored with diaminobenzidine (Bioss-Bio Ltd.) at room temperature for 7 min. Finally, hematoxylin was applied for 3 min to counterstain the sections which were then dehydrated, rinsed and coverslipped with glycerin. Negative controls were treated in the absence of primary antibodies. Quantitative analysis was performed using an image analysis system. The system included MetaMorph image acquisition and processing software (JADA 801D; JEDA Science-Technology Development Co., Ltd., Nanjing, China) and a Nikon 80i microscope (Nikon, Tokyo, Japan) equipped with a HP computer. Layers were analyzed from various subfields of the dorsal hippocampus, including the stratum oriens (SO), stratum radiatum (SR) and stratum lacunosum-moleculare (SLM) in CA1; the SO, stratum lucidum (SL) and SR in CA3; and the polymorphic layer (PL) and molecular layer (ML) in the dentate gyrus (DG). An image of the complete hippocampal formation was obtained initially at a low magnification of ×40 and then images at a higher magnification of ×200, in various subfields of the hippocampus, were acquired according to the size of each subfield: three images in CA1 for SO and SR; one image in CA3 and DG-PL; and two images in DG-ML and CA1-SLM. Digital data were exported into MetaMorph software for analysis and processing. The average optical density (OD) represented the intensity of immunohistochemical staining.

### Statistical analysis

Data were analyzed using SPSS 17.0 for Windows (SPSS, Inc., Chicago, IL, USA) and are presented as mean ± SEM. One way analysis of variance, using least-significant difference for post hoc analysis, was used to determine the total serum concentrations of T3, T4 and TSH, as well as the immunoreactivity of syntaxin-1 and munc-18 for all treatment groups. P<0.05 was considered to indicate a statistically significant difference.

## Results

### Serum concentrations of the hormones

Serum T3, T4 and TSH concentrations are presented in Table I. The serum T3 and T4 levels were significantly lower (P<0.01) and TSH levels were significantly higher (P<0.01) in the SD rats of the Hypo and DON groups than in those in the C group. T4 and T4 + DON treatment restored T3, T4 and TSH levels to values that were not significantly different from those in the control group (P>0.05).

### Protein levels of syntaxin-1 and munc-18 in the hippocampus

**Figure 1:**
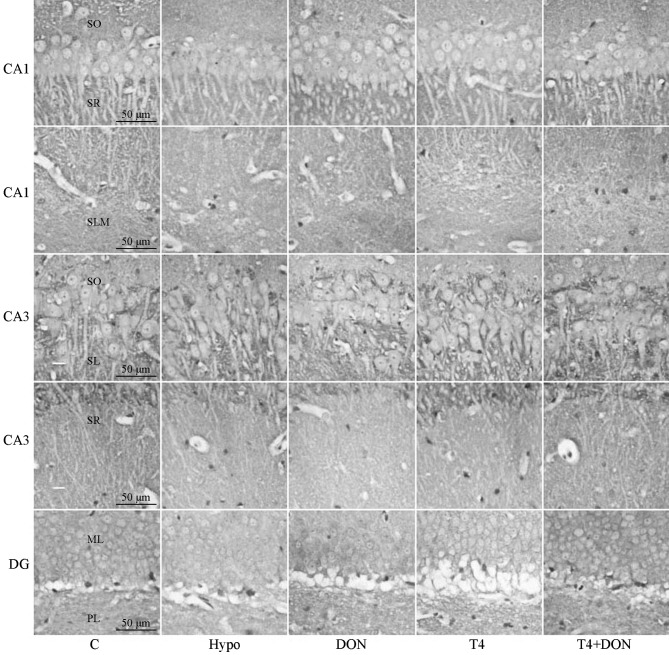
Photomicrographs of coronal sections showing munc-18 immunoreactivity in CA1, CA3 and DG subregions of the hippocampi of rats from the Hypo, T4, DON, T4 + DON and C groups (n=10–12). Distinct punctate spots of reaction product were observed in every layer of CA1, CA3 and DG subregions; note a slight reduction in overall staining intensity of CA3-SR, DG-ML and DG-PL in the Hypo, DON and T4 groups (magnification, ×400; scale bar, 50 μm). C, control group; Hypo, hypothyroid group; DON, hypothyroid rats treated with 0.005% (w/v) DON in drinking water; T4, hypothyroid rats treated with 6 μg T4/100 g BW; T4 + DON, hypothyroid rats treated with 6 μg T4/100 g BW plus 0.005% (w/v) donepezil in drinking water. SO, stratum oriens; SR, stratum radiatum; SLM, stratum lacunosum-moleculare; SL, stratum lucidum; ML, molecular layer; PL, polymorphic layer; T4, thyroxine; DON, donepezil; BW, body weight.

**Figure 2:**
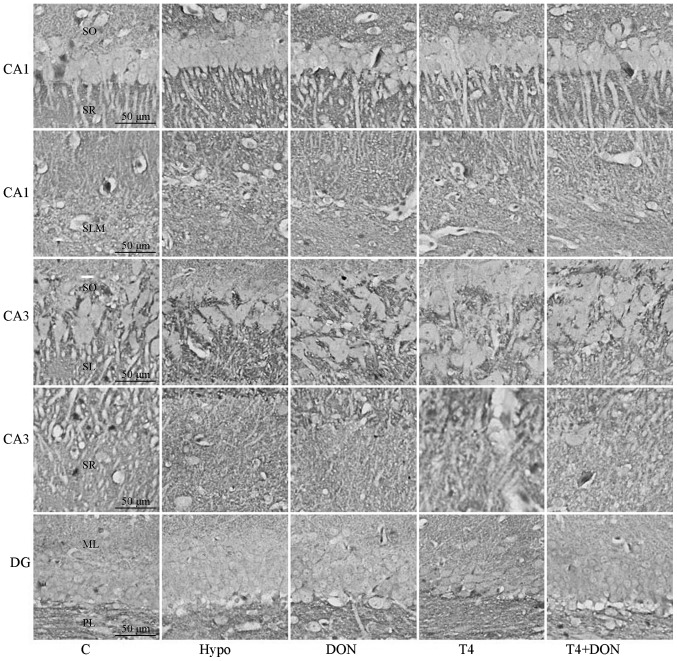
Photomicrographs of coronal sections showing syntaxin-1 immunoreactivity in CA1, CA3 and DG subregions of the hippocampi of rats from the Hypo, T4, DON, T4 + DON and C groups (n=10–12). Distinct punctate spots of reaction product were observed in every layer of CA1, CA3 and DG subregions; note that the staining for syntaxin-1 was more intense in DG-PL and in all layers of CA1 and CA3 of Hypo and DON groups and that the overall staining intensity was equal in the DG-ML of each of the five groups (magnification, ×400; scale bar, 50 μm). Hypo, hypothyroid group; DON, hypothyroid rats treated with 0.005% (w/v) DON in drinking water; T4, hypothyroid rats treated with 6 μg T4/100 g BW; T4 + DON, hypothyroid rats treated with 6 μg T4/100 g BW and 0.005% (w/v) DON in drinking water; C, control group. SO, stratum oriens; SR, stratum radiatum; SLM, stratum lacunosum-moleculare; SL, stratum lucidum; ML, molecular layer; PL, polymorphic layer; DON, donepezil; T4, thyroxine; BW, body weight.

Representative photomicrographs of the immunolabeled munc-18 and syntaxin-1 proteins in the different groups are presented in Figs. 1 and 2, respectively. The distributions of syntaxin-1 and munc-18 in the dorsal hippocampus were similar among the five groups. Each layer in the CA1, CA3 and DG subfields exhibited punctate spots of reaction product and the CA3-SL subfield was observed to exhibit large spots of munc-18, where large terminals of mossy fiber were located (Figs. 1 and 2).

Tables II and III present the analyzed OD values of munc-18 and syntaxin-1 immunoreactivity in each stratum of the hippocampal subfields. The OD values of munc-18 in three layers of the CA3 and DG subfields, i.e., CA3-SR, DG-PL and DG-ML, in the Hypo, DON and T4 groups were significantly lower compared with those of the corresponding layers in the C group (P<0.05). In the T4 + DON group, the OD values in all layers were similar to those in the C group (P=0.170, 0.863 and 0.600 respectively). The OD values of syntaxin-1 in all layers of CA1 and CA3, and in DG-PL were observed to be significantly higher in the Hypo group compared with those in the corresponding layers in the C group (P<0.01). No significant differences were identified between the T4 group and the C group, but the absolute values of the OD of syntaxin-1 in the T4 group were larger than those of the control (P>0.05). In the T4 + DON group, the OD values in these layers were more similar to those in the C group (P>0.05).

### Content of ACh in the hippocampus

**Figure 3:**
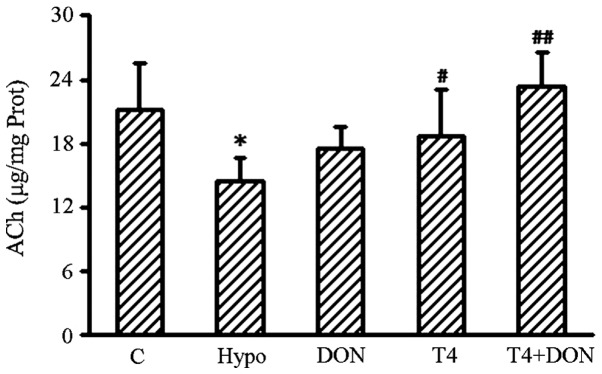
Concentration of hippocampal ACh in the Hypo, T4, DON, T4 + DON and C groups (n=10–12). Homogenates were extracted from the hippocampus of each rat. Hypothyroidism induced a significant reduction in ACh content in the hippocampus and the DON (0.005%), thyroxine (T4; 6 μg/100 g BW) or combined treatment (T4 + DON) restored the ACh levels to control values. Data shown represent mean ± SEM of three independent experiments. Hypo, hypothyroid group; DON, hypothyroid rats treated with 0.005% (w/v) DON in drinking water; T4, hypothyroid rats treated with 6 μg T4/100 g BW; T4 + DON, hypothyroid rats treated with 6 μg T4/100 g BW and 0.005% (w/v) DON in drinking water; C, control group. ^*^P<0.05, vs. control group; ^#^P<0.05 and ^##^P<0.01, vs. hypothyroid group. ACh, acetylcholine; T4, thyroxine; DON, donepezil; BW, body weight.

Alkaline hydroxylamine colorimetry was performed to detect the content of ACh in the hippocampi of the rats in the different groups. The ACh content in the hippocampus is illustrated in Fig. 3. The results show that the amount of ACh was significantly decreased by 24% in the hypothyroid rats (P=0.016) and the content was observed to be restored to control values by treatment with DON, T4 or T4 + DON (P=0.382, 0.265 and 0.411, respectively).

## Discussion

In the present study, immunohistochemical analysis revealed that the expression of munc-18 and syntaxin-1 was significantly altered in the hippocampus of adult-onset hypothyroid rats compared with that in the controls. Munc-18 in the Hypo group was expressed at a significantly lower level in the SR of CA3 and in the DG in the hippocampus. The results obtained in this study are consistent with a previous study reporting decreased munc-18 levels in the dorsal hippocampus of rats with adult-onset hypothyroidism (13). As it has been confirmed that TH regulates protein synthesis in the brain (19), the reduced expression of munc-18 may be associated with the lower TH neuronal levels in the hippocampus associated with hypothyroidism. Under the same conditions, the present study also observed that syntaxin-1 levels in the dorsal hippocampus were increased. Previous studies have shown that the expression of syntaxin-1 is upregulated in the adrenal gland in rats with secondary hypothyroidism, induced by hypophysectomy (20). In thyroidectomized rats, levels of syntaxin-1 have been shown to be downregulated in the adenohypophysis (21) and reductions in the expression of syntaxin-1 were also observed in the prefrontal cortex of rats with PTU-induced hypothyroidism (22). The regulation mechanism of syntaxin-1 is unknown. Previous studies have indicated that hypothyroidism induces various quantitative distributions of THs (23), as well as unidentically changing the isoforms of the thyroid receptor (TR) in various regions of the brain; for example, in the hippocampus and cerebral cortex, the relative expression of TRα1 was shown to increase, whereas the expression of TRα2 was decreased (24). It is possible that various TR isoforms, in different nervous tissues, regulate syntaxin-1.

In the current study, a significant decrement of ACh content in the hippocampus of adult-onset hypothyroid rats was observed. Decreased ACh content has also been identified in the spinal cords of methimazole-induced adulthood hypothyroid rats (25). It has been reported that a deficit in THs yields cholinergic neurons with a small somata and decreased numbers (26), thus leading to an insufficient synthesis of neurotransmitters. In addition, evidence from tissue culture experiments indicates that the enzymes responsible for the synthesis of ACh are under direct TH control (27); in the absence of THs, the enzymatic activity is weakened, hence the synthesis of ACh is decreased. In the current study, DON treatment ameliorated the reduction of ACh content in the Hypo group. This phenomenon also occurs when cholinesterase inhibitors, including DON, neostigmine and galantamine, are used to treat other diseases; for example, with the oral administration of DON to treat mild cognitive impairment (16,17) and the oral or intramuscular injection of neostigmine treatment for myasthenia gravis (28). The mechanism of this phenomenon is consistent with the hypothesis that cholinesterase inhibitors increase ACh levels by preventing the enzymatic degradation of ACh, thus prolonging its availability (15,29). In addition, galantamine and other AChE inhibitors may act as agonists at nicotinic receptors and enhance the release of ACh via a nicotinic mechanism, particularly under conditions of impaired cholinergic function (30).

T4 replacement therapy has been shown to re-establish plasma TH euthyroidism in adult-onset hypothyroidism, thereby attenuating reductions in the levels of ACh (31,32) and the impaired expression of synaptic proteins associated with cognitive function (33). In the present study, the synaptic proteins syntaxin-1 and munc-18 were restored by T4 replacement therapy; however, munc-18 levels did not reach those in the control. Previous animal studies have also reported that normal ranges of hormone substitution restored CaMKII, calmodulin, SNAP-25 and neurogranin but not protein kinase C-γ and syt-1 in hypothyroid rats (1,13), indicating that T4 replacement therapy causes asynchronous recovery of adult-onset hypothyroidism-induced molecular impairments in the brain. The asynchronous recovery may be associated with the different distributions and properties of these proteins in neurons (34). With regard to the failure to fully restore munc-18 expression, it is possible that the recovery of munc-18 in the hypothyroid hippocampus requires a different dose of exogenous T4. Studies using the isotopic equilibrium technique identified that the concentration of T4 in plasma greatly exceeded that present in the central nervous system (35). Despite T4 replacement therapy enabling serum THs to reach euthyroidism, the hormone substitution in the brain may still be insufficient. Indeed, this concept is supported by a study in which munc-18 in the brain was fully restored by a large dose of T4 (20 μg/100g BW) (13). However, large doses of T4 therapy result in marked increases in serum TH leves that may be detrimental to health. Therefore, the present study explored the effect of DON upon hypothyroidism.

In the DON + T4 group, munc-18 was found to be restored to control values in all layers, although the exact mechanisms underlying this regulation remain uncharacterized. Accumulated evidence from previous studies suggests that DON possesses neuroprotective properties in the suppression of neurodegeneration (15,36). Studies have reported that the neuroprotective effects of DON slow the progression of hippocampal atrophy in Alzheimer’s disease (37), protect cortical neurons in models of oxygen-glucose deprivation and glutamate-induced toxicity, protect against the effects of hippocampal mitochondrial dysfunction in transgenic mouse models (38), and increase the total dendritic length and spine density of neurons in aged mice (39). In addition, DON treatment has been shown to be effective in preserving presynaptic protein in the hippocampus and spinal cord in a tauopathy mouse model (40). Although the mechanisms concerning the neuroprotective effect of DON are not currently explicit, the neuroprotection observed upon the administration of DON is unlikely to be associated with AChE inhibition, as the neuroprotection afforded by DON is not achieved by other cholinesterase inhibitors, including neostigmine, galantamine or rivastigmine (38). DON may induce its neuroprotective effect by activating the neurotrophin receptors in the hippocampus (41). In addition, it has been shown that DON protects neurons by upregulating nicotinic acetylcholine receptor subtypes to decrease the glutamate toxicity that is involved in a number of neuronal degenerative diseases (36,39,42). In this context, the recovery of synaptic protein munc-18 in the co-administration group may occur as a result of DON-induced neuroprotection against hippocampal neuronal impairment, leading to an altered synthesis of the synaptic proteins.

In conclusion, the present study showed that adult-onset hypothyroidism induced alterations of munc-18, syntaxin-1 and ACh levels in the hippocampus. The expression of syntaxin-1 and ACh content was restored by T4 monotherapy while the expression of munc-18 was not. Co-administration of T4 and DON resulted in more effective restorations than either alone. The thyroid hormone has a direct effect on metabolism of hippocampal ACh in adult rats, and DON is helpful for treatment of synaptic protein impairment induced by hypothyroidism. Further research is required to investigate the efficacy of DON treatment and the molecular mechanism underlying this regulation, particularly, the long-term effects of acetylcholinesterase inhibitors on behavior and synaptic proteins in mouse models of hypothyroidism.

## Figures and Tables

**Table I:** Serum T3, T4 and TSH levels in the five groups.

Group	Number	T3, nmol/l	T4, nmol/l	TSH, μIU/ml
C	12	0.83±0.03	49.81±1.08	1.02±0.14
Hypo	11	0.60±0.03^a^	18.19±1.72^a^	19.78±3.01^a^
DON	11	0.57±0.02^a^	18.58±0.91^a^	19.55±3.29^a^
T4	10	0.83±0.08	52.42±1.92	1.21±0.32
T4 + DON	11	0.77±0.07	52.71±2.04	1.07±0.15

Data are expressed as the mean ± SEM. C, control group; Hypo, hypothyroid group; DON, hypothyroid rats treated with 0.005% (w/v) DON in drinking water; T4, hypothyroid rats treated with 6 μg T4/100 g BW; T4 + DON, hypothyroid rats treated with 6 μg T4/100 g BW and 0.005% (w/v) DON in drinking water.

a P<0.01, vs. control group.

T3, triiodothyronine; T4, thyroxine; TSH, thyroid-stimulating hormone; DON, donepezil; BW, body weight.

**Table II:** Munc-18 expression in various layers of each subfield in the hippocampus.

Subfield	Stratum	C	Hypo	DON	T4	T4 + DON
CA1	SO	4.63±0.90	4.43±0.88	4.43±1.02	4.53±0.66	4.67±0.97
	SR	3.82±0.85	3.53±0.56	3.61±0.66	3.69±0.97	3.91±0.77
	SLM	4.37±0.76	3.93±0.87	4.06±0.74	4.09±0.76	4.21±0.78
CA3	SO	4.62±0.72	3.63±0.72	3.91±0.88	4.35±0.68	4.68±0.70
	SL	3.55±0.88	3.12±0.79	3.19±0.60	3.21±0.67	3.67±0.77
	SR	4.88±0.76	3.42±0.53^b^	3.69±0.63^b^	4.19±0.55^a^	4.59±0.69
DG	ML	4.61±0.70	3.34±0.93^b^	3.61±1.31^b^	3.80±0.91^a^	4.67±0.59
	PL	4.11±0.50	3.31±0.78^b^	3.42±0.46^b^	3.54±0.64^a^	4.26±0.65

Data are presented as mean ± SEM and expressed as the average OD of munc-18 immunoreactivity (n=10–12). C, control group; Hypo, hypothyroid group; DON, hypothyroid rats treated with 0.005% (w/v) DON in drinking water; T4, hypothyroid rats treated with 6 μg T4/100 g BW; T4 + DON, hypothyroid rats treated with 6 μg T4/100 g BW and 0.005% (w/v) DON in drinking water.

a P<0.05 and

b P<0.01, vs. the control group.

DG, dentate gyrus; SO, stratum oriens; SR, stratum radiatum; SLM, stratum lacunosum-moleculare; SL, stratum lucidum; ML, molecular layer; PL, polymorphic layer; OD, optical density; DON, donepezil; T4, thyroxine; BW, body weight.

**Table III:** Syntaxin-1 expression in various layers of each subfield in the hippocampus.

Subfield	Stratum	C	Hypo	DON	T4	T4+DON
CA1	SO	0.36±0.11	1.15±0.38^b^	1.19±0.29^b^	0.39±0.10	0.36±0.09
	SR	0.45±0.50	1.32±0.17^b^	1.24±0.33^b^	0.49±0.10	0.45±0.78
	SLM	0.42±0.13	1.13±0.41^b^	0.96±0.29^b^	0.48±0.14	0.40±0.11
CA3	SO	0.89±0.14	1.21±0.32^b^	1.14±0.35^a^	1.00±0.33	0.90±0.25
	SL	1.02±0.19	1.55±0.59^b^	1.20±0.14^b^	1.07±0.20	1.01±0.11
	SR	0.71±0.95	1.31±0.33^b^	1.23±0.43^b^	0.73±0.12	0.70±0.14
DG	ML	1.70±0.67	1.91±0.60	1.77±0.42	1.81±0.62	1.55±0.67
	PL	2.06±0.49	2.87±0.53^b^	2.76±0.37^b^	2.12±0.36	2.07±0.44

Data are presented as mean ± SEM and expressed as the average OD of syntaxin-1 immunoreactivity (n=10–12). C, control group; Hypo, hypothyroid group; DON, hypothyroid rats treated with 0.005% (w/v) DON in drinking water; T4, hypothyroid rats treated with 6 μg T4/100 g BW; T4 + DON, hypothyroid rats treated with 6 μg T4/100 g BW and 0.005% (w/v) DON in drinking water.

a P<0.05 and

b P<0.01, vs. the control group.

DG, dentate gyrus; SO, stratum oriens; SR, stratum radiatum; SLM, stratum lacunosum-moleculare; SL, stratum lucidum; ML, molecular layer; PL, polymorphic layer; OD, optical density; T4, thyroxine; DON, donepezil; BW, body weight.
